# Enhancing tuberculosis treatment success: gender dynamics and designated drug supervisors in Tasikmalaya, Indonesia

**DOI:** 10.4314/ahs.v26i1.4

**Published:** 2026-03

**Authors:** Endang Puji Astuti, Mutiara Widawati, Tri Wahono, Yuneu Yuliasih, Wawan Ridwan, Lukman Hakim, Mara Ipa

**Affiliations:** 1 Research Center for Public Health and Nutrition, National Research and Innovation Agency, Cibinong, West Java, Indonesia; 2 Faculty of Medicine, Public Health and Nursing (FK-KMK), Gadjah Mada University, Yogyakarta, Indonesia

**Keywords:** Tuberculosis Management, Drug Supervision, Gender Disparities, Medication Adherence, Community Health Education, Tasikmalaya

## Abstract

**Background:**

Tuberculosis control in Tasikmalaya relies on competent drug supervisors (DS) to ensure adherence, yet knowledge gaps remain.

**Objectives:**

To assess factors influencing DS effectiveness in Tasikmalaya, Indonesia.

**Methods:**

A cross-sectional study of 121 DS was conducted. Data on sociodemographic factors, knowledge, attitudes, and practices were collected through validated questionnaires. Logistic regression was used to identify determinants of DS performance.

**Results:**

Female and officially designated DS were significantly more effective in ensuring adherence compared to males and voluntary DS. Overall knowledge was inadequate among most DS (95.9%). Cadres performed better than family members in supervisory tasks.

**Conclusion:**

Improving DS capacity—especially family and male supervisors—through structured training and official designation can enhance TB treatment outcomes.

## Introduction

Since the World Health Organization (WHO) declared Tuberculosis (TB) a global emergency in 1993, it remains a heavy burden, with the 2019 report documenting 11 million new cases and 242,000 deaths, worsened by COVID-19^1^,^2^. Meanwhile, Indonesia accounts for nearly two-thirds of all TB cases worldwide (8.5%)^1^,^2^. Indonesia, contributing significantly to this burden, projected 824,000 cases and 15,186 deaths in 2021. To combat this, Indonesia adopted the End TB strategy, aiming for TB elimination by 2030 with enhanced detection and a 90% treatment coverage goal, per WHO's DOTS (Directly Observed Treatment Short-course)^3^,^4^.

The success of tuberculosis treatment is influenced by various factors, including the role of drug supervisors (DS), medication adherence, drug availability, family support, and patient characteristics such as age, gender, education, and income 5,6. DS, known as Petugas Menelan Obat (PMO) in Bahasa Indonesia, are crucial for ensuring medication adherence, monitoring side effects, overseeing the treatment process, and providing patient support^5^. Their presence significantly improves patient adherence to treatment, thereby preventing drug resistance and transmission^6^,^7^.

Studies in Indonesia have linked DS involvement to higher TB therapy success^8^-^10^. This underscores the importance of DSs' trustworthiness, patient proximity, and support willingness. However, knowledge gaps among DSs could hinder their treatment oversight effectiveness^11^-^13^. Effective TB treatment relies on the combined knowledge and satisfaction of both patients and supervisors, making the selection of DS crucial, especially in high TB areas like Tasikmalaya.

Tasikmalaya, a city in West Java, is one such area with a high risk of TB. In 2019-2020, the city reported 1,531 new TB cases, with a success rate (SC) fo 90% and a cure rate (CR) of 26%. In 2020, there were 935 new cases with an SC of 90% and a CR of 39%^14^. Despite these high numbers and continuous efforts in implementing the End TB strategy involving DS, the determinants affecting DS performance in such regions are still not well understood. Understanding these determinants is crucial for improving TB treatment outcomes. Therefore, this study aims to fill this gap by investigating the factors influencing the effectiveness of DS in Tasikmalaya.

## Method

### Study Design

This study is a cross-sectional analysis conducted to evaluate the performance of Drug Supervisors (DS) in supervising tuberculosis (TB) treatment in Tasikmalaya, West Java, Indonesia. A cross-sectional design was selected because it allows simultaneous assessment of socio-demographic factors, knowledge, attitudes, and supervisory practices, providing a snapshot of determinants influencing DS performance in Tasikmalaya.

### Study setting

This study was conducted in Tasikmalaya, West Java, Indonesia. Data collection took place over a two-month period, from June to August 2021 targeting areas with high TB prevalence. The study covered nine Primary Health Centers (PHCs), including Panglayungan and Cipedes. A total of 121 DS, who were active between 2019 to 2021, participated in the study. Rather than using a formal sample size calculation, a ratio-based approach was applied to ensure proportional representation of drug supervisors across the nine participating health centers.

### Data Collection

All individuals serving as drug supervisors during 2019–2021, regardless of age, were eligible for participation. The youngest DS at the time of data collection was 15 years old. Data were collected via a validated questionnaire. The questionnaire was adapted from existing TB KAP instruments and further developed by the research team to reflect the local context. Face validity was established through expert review (two TB specialists, one community health expert). A pilot test with 20 DS outside the study site was conducted to assess clarity and cultural appropriateness. Reliability was assessed using test–retest within two weeks, yielding Cronbach's alpha values ranging from 0.72 to 0.84, indicating acceptable internal consistency.

**The questionnaire was divided into four main sections**

1. Socio-demographic details: This section gathered information about the DS and patients, including age, gender, education level, employment status, marital status, TB treatment stages and BCG vaccination status.
2. Knowledge Assessment: A 15-item knowledge assessment focused on TB knowledge, covering topics such as TB transmission, symptoms, treatment, and prevention methods
3. Perception Survey: A 15-item survey to assess DS's perceptions of their impact on treatment outcomes, including their views on the importance of their role and their confidence in their knowledge.
4. Task Adherence Evaluation: A 16-item evaluation of DS's adherence to their tasks, such as supervising medication intake, monitoring side effects, and providing patient support.

Responses were captured through multiple-choice and Likert scale ratings. A scores of ≥75% was considered proficient.

### Variables

The primary outcome variable was the effectiveness of DS in supervising TB medication adherence. Independent variables included socio-demographic factors, knowledge scores, perception scores, and adherence scores of DS.

### Data Analysis

The collected data were initially entered into Microsoft Excel for cleaning and preprocessing., Subsequently, the data were analyzed using SPSS version 17. Descriptive statistics were used to summarize the socio-demographic characteristics and other variables. Logistic regression analyses were conducted to identify the factors influencing DS performance, with statistical significance set at p<0.05.

## Ethical consideration

The study received ethical approval from the National Institute of Health Research and Development of Indonesia (Ref. LB.02.01/2/KE.155/2021). Participation in the study was voluntary. Informed consent was obtained from all participant, and privacy was ensured through the use of unique identifiers for each subjects.

## Result

### Characteristic of TB drug supervisors

Table 1 shows 73.6% of Tasikmalaya's 121 TB drug supervisors are family members; while the remaining 26.4% are TB cadres. Predominantly, these supervisors are older than 45, female, married (89.9%), unemployed (75.2%), and have a high school education (69.4%). Their supervision typically spans 1-9 months, with roles assigned either as voluntaryor officially designated DS. A significant knowledge gap on TB treatment stages exists, more so among family supervisors (82.1%) than cadres (43.8%), with widespread cigarette exposure in patients' households (31.4%). Overall, supervisors' TB knowledge is low (95.9%), particularly among family members (98.8%). Despite this, cadres show exemplary performance (100%) in supervision tasks, contrasting with family members (52.8%), who show a notable lag, indicating a critical area for intervention to enhance TB treatment outcomes.

**Table 1:** Sociodemographic characteristics and KAP of TB Drug Supervisors in Tasikmalaya, 2021

Characteristics	Family Members (n=89, 73.6%)	TB Cadres (n=32, 26.4%)	Total (n=121)
**Age Groups (years)**			
15-24	9 (10.1%)	0 (0%)	9 (7.4%)
25-44	35 (39.3%)	14 (43.75%)	49 (40.5%)
≥45	45 (50.6%)	18 (56.25%)	63 (52.1%)
**Gender**			
Male	19 (21.3%)	0 (0%)	19 (15.7%)
Female	70 (78.7%)	32 (100%)	102 (84.3%)
**Current Marital Status**			
Single	9 (10.1%)		9 (7.4%)
Currently Married	76 (85.4%)	31 (96.9%)	107 (88.4%)
Formerly Married	4 (4.5%)	1 (3.1%)	5 (4.1%)
**Employment**			
Unemployed	64 (71.9%)	27 (84.4%)	91 (75.2%)
Employed	25 (28.1%)	5 (15.6%)	30 (24.79%)
**Educational status**			
Primary school	27 (30.3%)	4 (12.5%)	31 (25.6%)
High school	60 (67.4%)	24 (75%)	84 (69.4%)
University degree	2 (2.2%)	4 (12.25%)	6 (5.0%)
**Duration as DS (tenure)**			
1-9 month	82 (92.1%)	27 (84.4%)	109 (90.1%)
>9 month	6 (6.7%)	5 (15.6%)	11 (9.9%)
**Appointment as a DS**			
Voluntary	58 (65.2%)	3 (9.4%)	61 (50.4%)
Officially designated	31 (34.8%)	29 (90.6%)	60 (49.6%)

**History of TB patient's**			

**Treatment of drug-susceptible** **TB**			
Intensive phase	4 (4.50%)	8 (25.0%)	12 (9.91%)
Continuation phase	2 (2.20%)	3 (9.37%)	5 (4.13%)
Both	10 (11.2%)	7 (21.9%)	17 (14.0%)
Don't know	73 (82.0%)	14 (43.7%)	87 (71.9%)
**Cigarette exposure**			
yes	54 (60.7%)	29 (90.6%)	83 (68.6%)
no	30 (33.7%)	3 (9.4%)	33 (27.3%)
Don't know	5 (5.6%)	0 (0%)	5 (4.1%)

**Knowledge, Attitude and Practice**			

**Knowledge**			
Understanding the transmission routes of TB	82 (92.1%)	32 (100%)	114 (94.2%)
Understanding different types of TB	44 (49.4%)	32 (100%)	76 (62.8%)
Understanding the causative agent of TB	8 (8.9%)	10 (31.3%)	18 (14.9%)
Understanding the symptoms of TB	73 (82.0%)	32 (100%)	105 (86.8%)
Understanding TB treatment drugs and their durations	7 (7.9%)	6 (18.7%)	13 (10.7%)
Understanding TB testing	71 (79.8%)	31 (96.9%)	102 (84.3%)
Understanding about TB drug side effects	59 (66.3%)	32 (100%)	91 (75.2%)
Understanding the responsibilities of a DS	73 (82.0%)	32 (100%)	105 (86.8%)
Adequacy of knowledge	1 (1.1%)	4 (12.5%)	5 (4.1%)
Inadequacy of knowledge	88 (98.8%)	28 (87.5%)	116 (95.87%)
**Attitude**			
Supervising pulmonary TB patients for complete medication adherence	84 (94.4%)	31 (96.9%)	115 (95.0%)
Continuing medication despite feeling improvement.	71 (79.8%)	30 (93.7%)	101 (83.5%)
Requiring education on managing TB patients at home	76 (85.4%)	28 (87.5%)	104 (85.95%)
Requiring sputum examination to confirm pulmonary TB	65 (73.0%)	28 (87.5%)	93 (76.9%)
Adequacy of attitude	80 (89.9%)	32 (100.0%)	112 (92.6%)
Inadequacy of attitude	9 (10.1%)	0 (0%)	9 (7.4%)
**Practice**			
Being a supervisor in taking medicine	69 (77.5%)	27 (84.4%)	96 (79.3%)
Conveying side effects	53 (59.6%)	31 (96.9%)	84 (69.4%)
Supervising control	65 (73.0%)	27 (84.4%)	92 (76.0%)
Providing motivation and enthusiasm	68 (76.4%)	31(96.9%)	99 (81.8%)
Adequacy of practice	42 (47.2%)	32 (100%)	74 (61.2%)
Inadequacy of practice	47 (52.8%)	0 (0%)	47 (38.8%)

### Logistic Regression Analysis

The present analysis focused on logistic regression to identify predictors of DS performance. Table 2 presents the logistic regression outcomes identifying factors affecting TB drug supervisors' management practices. The analysis covered gender, employment, education, marital status, tenure, appointment method, and TB knowledge and attitudes, finding significant correlations. Notably, gender and appointment method influenced management practices; male supervisors and voluntary DS were both being less likely to ensure patient medication adherence compared to female supervisors and officially designated DS, respectively (aOR=0.09, 95% CI=0.02-0.49, p=0.005 for gender; aOR=0.314, 95% CI=0.13-0.76, p=0.01 for appointment method). Other variables showed no significant effect post-adjustment.

**Table 2:** Univariate and Multivariate Logistic Regression Analysis of Associated Factors of Practice (Pengawasan Minum Obat TB) Among Drug Supervisors, Tasikmalaya, 2021

Variables	Univariate analysis		Mutivariate analysis	

	Crude-OR (95% CI)	p-value	Adjusted-OR (95% CI)	p-value
**Age Groups** (Ref = ≥ 45 years)				
15-24 years	0.526 (0.129-2.152)	0.372		
25-44 years	1.238 (0.570-2.688)	0.589		
**Gender** (Ref = Female)				
Male	0.122 (0.037-0.397)	<0.001***	0.09 (0.02-0.49)	0.005**
**Current Marital Status** (Ref = Formerly Married)				
Single	1.200 (0.130-11.05)	0.872		
Currently Married	2.615 (4.19-16.34)	0.304		
**Employment** (Ref = Employed)				
Unemployed	2.212 (0.957-5.114)	0.063	0.685 (0.199-2.358)	0.548
**Educational status** (Ref = University degree)				
Primary school	NS	0.999	NS	0.999
High school	NS	0.999	NS	0.999
**Duration as a DS** (Ref = >9 months)				
1-9 months	0.142 (0.018-1.15)	0.067	0.101 (0.01-1.05)	0.055
**Appointment as DS** (Ref = **Officially Designated)**				
Voluntary	0.409 (0.192-0.868)	0.02*	0.314 (0.130-0.76)	0.01*
**Status** (Ref = Cadres)				
Family/Community	NS	0.998	NA	NA
**TB Knowledge** (Ref = Adequate)				
Inadequate)			NA	NA
**TB Attitude** (Ref = Adequate)				
Inadequate)	0.159 (0.031-0.801)	0.026*	0.216 (0.02-1.415)	0.110

* p<0.05

** p<0.01

*** p<0.001

NS: Not Significant

NA: Not Applicable (were not included into the multivariate analysis)

## Discussion

The role of Tuberculosis (TB) drug supervisors (DS) is crucial in improving TB treatment outcomes. Research shows that having a DS significantly enhances treatment success rates and patient adherence to medication regimens 5,6. Our study highlights that gender and formal designation of DS significantly impact DS performance and TB treatment success. Female drug supervisors, particularly married women, often perform better due to their caregiving tendencies and attention to detail^15^,^16^. Male drug supervisors, on the other hand, were found to be less effective in assisting TB patients.

Official drug supervisor appointments, as opposed to volunteer support, underline the importance of commitment in patient adherence^17^,^18^. Formal appointments are more structured and allow for easier monitoring and evaluation. Directly Observed Therapy (DOT) by drug supervisors has been shown to result in higher treatment success rates, higher sputum conversion rates, and lower treatment failure and dropout rates^18^.

Drug supervisor backgrounds, whether as healthcare professionals or family members, are crucial in supporting TB patients due to their close relationships and trustworthiness^19^,^20^. Effective communication between supervisors and patients is recommended to motivate patients and improve therapy success rates^31^. Additionally, the presence of drug supervisors provides psychological support, encouraging TB patients to complete their therapy. In Tasikmalaya, there is a preference for family-based care, with only 26.1% of TB patient Drug Supervisors being health cadres. Despite a preference for family-based care, our findings suggest that health cadres, if properly trained, can significantly contribute to TB care^12^,^21^-^23^.

While educational and professional backgrounds matter^24^, experiential and cognitive factors are key to DS effectiveness^25^. Our study found that education, occupation, duration of service as DS, and attitude were significantly associated with DS performance in supervising TB patient treatment, while knowledge alone had little impact. The very low knowledge levels among family supervisors (98.8% inadequate) may reflect their limited formal education, absence of structured TB training, and reliance on informal learning. In Tasikmalaya, family members often serve as DS by necessity rather than training, leading to knowledge gaps despite positive attitudes. This suggests that attitudes and behaviors might be influenced by factors other than knowledge, such as experience and cognitive abilities^25^. Strengthening family-based supervisors can be achieved through structured training and regular refresher workshops facilitated at the primary health center level. Such programs would not only increase technical knowledge but also build supervisory confidence, ultimately improving treatment adherence.

External barriers to adherence, such as unclear treatment objectives, long treatment period, side effects^26^-^28^, logistical issues^28^,^29^, poor patient-provider relationships^30^, and higher private healthcare costs^29^ highlight the need for comprehensive family and DS engagement. Emotional, practical, and consistent family support is essential for sustaining patient motivation and ensuring recovery^32^. This support includes reminding patients to take their medication, accompanying them to health facilities, and providing emotional and practical assistance during difficult times. Understanding the DS role enables families to provide exceptional support, enhancing patient adherence and improving quality of life^23^.

## Limitations

Despite providing key insights, this study has limitations. Sampling bias and reliance on self-reported data may affect generalizability and accuracy due to potential biases such as social desirability. While it sheds light on drug supervisors' knowledge, attitudes, and practices in TB treatment, it does not consider patient and healthcare system factors. However, it offers valuable perspectives on drug supervisor performance in TB management, encouraging further research and enhancements in control strategies.

## Conclusions

This study identified gender and appointment status as key determinants of DS performance in Tasikmalaya. Female and officially designated DS were more effective, while family-based and male supervisors showed significant gaps. Efforts should be focused on boosting knowledge and practices, especially among male DS, and favoring supervisor appointments to enhance TB outcomes. These findings highlight the need for structured training, official designation, and empowerment of family supervisors to strengthen TB treatment success in Indonesia.. Future research with a broader, varied sample and longitudinal data, including more variables, could strengthen option in improving TB control strategies.
